# Dr. R. M. Gage

**Published:** 1875-09

**Authors:** A. P. Merrill

**Affiliations:** Acting Secretary.


### OBITUARY.

*Dr. R. M. Gage.—Died July* 26th, 1875.—New York, July 31st, 1865.—A special meeting of "The American Academy of Dental Surgery" was held this evening to do honor to the memory of our worthy fellow, Dr. R. M. Gage.

The President, Dr. Geo. H. Perine, then stated the object of the meeting, and gave a brief sketch of the professional life of our deceased Fellow.

He stated "The Academy had been called upon to mourn the loss of one of its most respected and beloved Fellows, Dr. R. M. Gage, at the time of his death, Vice-President of the American Dental Convention.

"This sad occurrence took place at his residence, No. 47 West 49th Street, in this city, on the 26th July.

"Dr. Gage was born in Bedford, N. H., 1822, and at an early age developed a love for his profession, which he successfully pursued up to the time of his death.

"The funeral services were held in the presence of a large assemblage of his friends and professional brethren. Dr. Gage leaves a widow and two sons, one of whom is pursuing the practice of medicine.

"In conclusion, I desire to add my tribute to his worth. I knew him long and well, and I know that I feebly reflect your own thoughts in saying that I cannot but feel that his record was pure and untarnished; that he was a Christian man, a courteous gentleman, ever affable and genial, noble minded, generous and forgiving—always faithful to life's duties, he was an honest man, a good dentist, and as such we deplore his loss, not only to our Academy and the Profession, but the social community in which he moved."

A committee was then appointed to prepare resolutions expressive of the sorrow felt by this Academy in the loss of our late brother, as well as the high esteem in which he was held by the Fellows, who submitted the following preamble and resolutions, which were unanimously adopted by the Academy:

"We have been called to view a Fellow and a brother practitioner stricken suddenly by death in the midst of his usefulness, and in the full maturity of manhood. Well may we mourn the loss of so good a man as Dr. R. M. Gage, and we desire to offer our tribute of respect, and to testify our estimation of his worth, Therefore be it

*Resolved,* That in his death The American Academy of Dental Surgery has lost one of its most valued Fellows, whose skill and untiring energies were devoted to its welfare and prosperity, and whose deportment and character gave dignity to our profession.

*Resolved,* That to his family we tender our most heartfelt sympathy, and though their loss be irreparable, pray that our Heavenly Father may sooth their great grief and bestow upon them the consolation of His providence and grace.

The committee also recommended the following. which was adopted:

*Resolved,* That an authenticated copy of the Preamble and Resolutions adopted by this Academy be forwarded to the family of our deceased worthy Fellow, and also to the Medical and Dental Journals.

[Attest,] A. P. Merrill, *Acting Secretary.*

